# Supplementary material for: Predicting visual function by interpreting a neuronal wiring diagram
Source: Nature. 2024 Oct 2;634(8032):113–23. doi: 10.1038/s41586-024-07953-5 (PMC11446822; doi:10.1038/s41586-024-07953-5)
Supplement: Supplementary file 6 — CRF and ERF predictions for individual TmY4 and TmY9 cells. Analogous to Supplementary Data 3, but for TmY target types. Shown are the top four monosynaptic pathways, the strongest pathway passing through each of the top ten intermediary types (ranking from Extended Data Fig. 7), and the trisynaptic pathway Tm1–TmY–Dm3–TmY (see the section entitled Prediction of spatial normalization). [file 41586_2024_7953_MOESM6_ESM.zip › DataS4/TmY9q/720575940617636308.pdf]

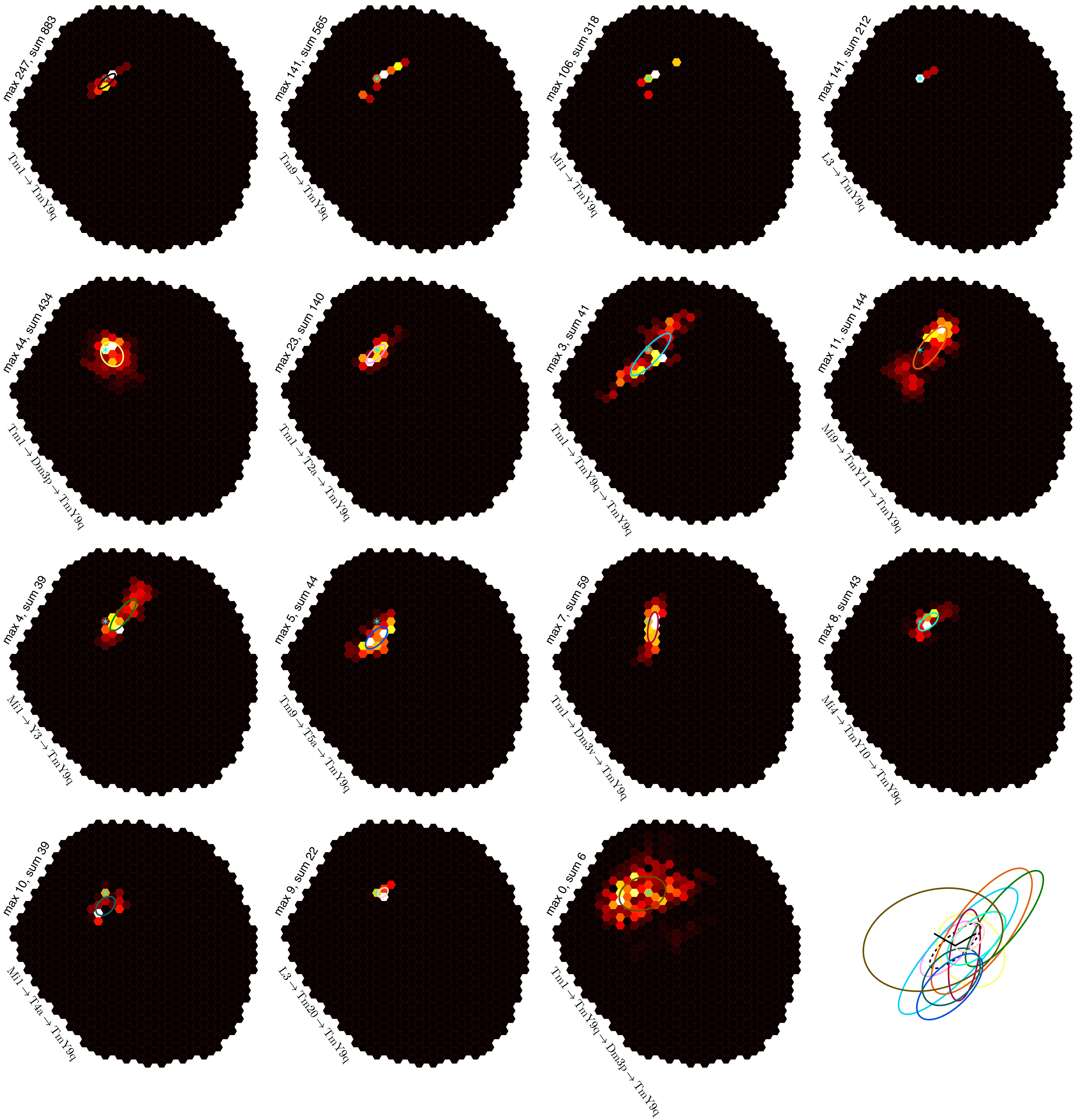

max 247, sum 883
Tm1 → TmY9q
max 141, sum 565
Tm9 → TmY9q
max 106, sum 318
Mi1 → TmY9q
max 141, sum 212
L3 → TmY9q
max 44, sum 434
Tm1 → Dm3p → TmY9q
max 23, sum 140
Tm1 → T2a → TmY9q
max 3, sum 41
Tm1 → TmY9q → TmY9q
max 11, sum 144
Mi9 → TmY11 → TmY9q
max 4, sum 39
Mi1 → Y3 → TmY9q
max 5, sum 44
Tm9 → T5a → TmY9q
max 7, sum 59
Tm1 → Dm3v → TmY9q
max 8, sum 43
Mi4 → TmY10 → TmY9q
max 10, sum 39
Mi1 → T4a → TmY9q
max 9, sum 22
L3 → Tm20 → TmY9q
max 0, sum 6
Tm1 → TmY9q → Dm3p → TmY9q
